# Compound-Specific Chlorine Isotope Analysis of Organochlorine Pesticides by Gas Chromatography-Negative Chemical Ionization Mass Spectrometry

**DOI:** 10.1155/2021/8874679

**Published:** 2021-01-28

**Authors:** Jing Zhang, Shenghua Liu, Jianye Gui, Xiaoya Li, Guochen Qi

**Affiliations:** ^1^Key Laboratory of Groundwater Sciences and Engineering, Ministry of Natural Resources, Institute of Hydrogeology and Environmental Geology, CAGS, Shijiazhuang 050061, China; ^2^Key Laboratory of Groundwater Remediation of Hebei Province and China Geological Survey, Shijiazhuang 050061, China; ^3^School of Earth Sciences and Resources, China University of Geosciences (Beijing), Beijing 100083, China

## Abstract

Compound-specific stable chlorine isotope analysis (CSIA-Cl) is an important method for identifying sources of organochlorine contaminants and helping assess their quantification of transformation processes. However, the present CSIA-Cl is challenged by either redundant conversion pretreatment or complicated mathematical correction. To overcome the mentioned problems, a novel method has been developed for the CSIA-Cl of eight organochlorine pesticides using gas chromatography-negative chemical ionization mass spectrometry (GC-NCI-qMS) in this study. The instrument parameters, acquisition mode, and required injection amounts were optimized in terms of the precision of GC-NCI-qMS. An ionization energy of 90 eV and emission current of 90 *μ*A were selected, and the precisions for eight organochlorine pesticides were in the range of 0.37‰–2.15‰ in single ion monitoring (SIM) mode when the injected amount was 0.50 mg L^−1^ (*viz*. 0.5 ng on column). Furthermore, when standards from Supelco and O2si were calibrated using standards from AccuStandard regarded as external isotope standard, chlorine isotope composition of *α*-hexachlorocyclohexane (*α*-HCH) and 2, 2-dichloro−1, 1-bis (4-chlorophenyl) ethylene (*p*, *p′*-DDE) in Supelco and O2si was confidently differentiated. The provenance identification method was validated by three organochlorine contaminated groundwater samples and showed a prospect in identifying the source of organochlorine pesticides.

## 1. Introduction

Various chlorinated organic compounds are widely used in many industries, while most of them have shown strong toxicity and persistence [1], and are found throughout the environment [2, 3]. Elucidating the sources and transformation processes of these organic pollutants and evaluating their possible impacts on humans and the environment are of great importance. In the past decades, compound-specific stable chlorine isotope analysis (CSIA-Cl) has been developed as an effective tool to identify sources of chlorinated organic compounds and assess their biodegradation processes and abiotic transformation [4–12].

Traditional CSIA-Cl methods require tedious offline or online sample treatment to convert the target chlorinated analytes into compounds containing only one chlorine atom (such as CsCl, CH_3_Cl, and HCl). These measurable compounds are then analyzed, such as CsCl by thermal ionization mass spectrometry (TIMS) [13, 14] and CH_3_Cl by dual inlet isotope ratio mass spectrometry (DI-IRMS) [15–17]. Shouakar-Stash et al. [18] first established an online method without a conversion process for the CSIA-Cl of chlorinated alkene using gas chromatography coupled with continuous flow isotope ratio mass spectrometry (GC-CF-IRMS), determining the lower limits of quantification (LOQ) and eliminating offline separation.

As the above instruments are expensive and difficult to access, regular GC-quadrupole mass spectrometry (GC-qMS) methods for CSIA-Cl are receiving increasing attention. Sakaguchi-Söder et al. [19] developed the first method for the CSIA-Cl of tetrachloroethene (PCE) and trichloroethene (TCE) by direct injection using a standard GC-qMS system. Although this method did not involve offline sample preparation, a set of mathematical equations were still required to calculate the chlorine isotope ratios. Accordingly, Aeppli et al. [20] established an improved measurement strategy and data evaluation using standard isotope bracketing. Jin et al. [21] also optimized the method, while Bernstein et al. [22] systematically compared the performance of GC-qMS and GC-IRMS between different types of instruments. The aforementioned methods calculated the chlorine isotope ratios, with isotopologues of the detected ions conforming to the binomial distribution, which was tacitly approved [19]. The results obtained from different ions may be diverse without calibration by isotope standards, while the chlorine isotopologues of some chlorinated organic compounds are unlikely to obey the binomial distribution in their product ions because of the chlorine isotope fractionation caused by GC-MS systems, especially electron ionization (EI) part [23].

Currently, the main problems of CSIA-Cl methods are as follows: (i) complex pretreatment and instruments limit multicomponent analysis online; (ii) isobaric interference cannot be completely overcome; and (iii) complicated correction formulas may be required. In this study, a novel scheme for ^37^Cl/^35^Cl ratio analysis has been established using gas chromatography-negative chemical ionization mass spectrometry (GC-NCI-qMS). This method was evaluated in terms of precision and amount dependency by optimizing the instrument parameters and acquisition mode. As no known chlorine isotope composition standard was available, different standards were considered as mutual external isotope standards to determine the chlorine source. Organochlorine-contaminated groundwater samples were analyzed by this novel CSIA-Cl method to validate the provenance identification by *δ*^37^Cl′ values.

## 2. Experimental

### 2.1. Chemicals and Materials

Three standard solution mixtures containing eight organochlorine pesticides, namely, *α*-hexachlorocyclohexane (*α*-HCH; >99.5%), *β*-hexachlorocyclohexane (*β*-HCH; >99.9%), *γ*-hexachlorocyclohexane (*γ*-HCH; >99.7%), *δ*-hexachlorocyclohexane (*δ*-HCH; >98.2%), 1-chloro-2-[2, 2, 2-trichloro−1-(4-chlorophenyl) ethyl] benzol (*o, p*′-DDT; >98.6%), 2,2-bis (*p*-chlorophenyl)−1, 1, 1-trichloroethane (*p, p′*-DDT; >99.9%), 2,2-dichloro−1, 1-bis (4-chlorophenyl) ethylene (*p, p*′-DDE; >99.4%), and 1, 1-dichloro-2, 2-bis (*p*-chlorophenyl) ethane (*p, p*′-DDD; >99.1%), were purchased from AccuStandard (New Haven, USA), Supelco (Bellefonte, PA, USA), and O2si Smart Solutions (South Carolina, USA), respectively. Chromatographic-grade n-hexane, methanol, ethyl acetate, and dichloromethane were purchased from Fisher Chemical (Fisher Scientific, USA). Each standard stock solution was subsequently diluted in n-hexane to working concentrations (0.05–1.00 mg L^−1^) using volumetric flasks and gas-tight glass syringes.

### 2.2. Sample Pretreatment

Water samples were extracted using solid-phase extraction (SPE). First, 0.2 L water was mixed with 10 mL methanol. C18 SPE column was activated with 5 mL ethyl acetate, 5 mL methanol, and 10 mL ultrapure water, holding the flow rate of 5 mL min^−1^. Subsequently, the water sample was passed through the SPE column at a flow rate of 10 mL min^−1^ and washed with 10 mL ultrapure water. The SPE column was eluted with 2.5 mL ethyl acetate and 5 mL dichloromethane at a flow rate of 5 mL min^−1^; then, the eluent was collected into the concentration tube. After dried by anhydrous sodium sulfate, the eluent was concentrated to 1 mL and then analyzed. More details could refer to the national environmental protection standards of the People's Republic of China (HJ 699-2014) [24].

### 2.3. Gas Chromatography-Negative Chemical Ionization Mass Spectrometry Analysis

**Figure 1:**
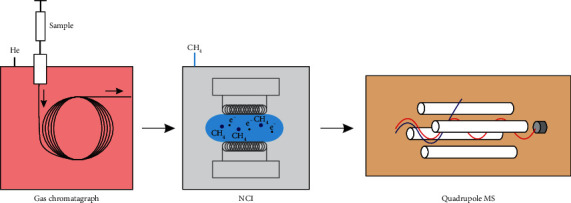
Schematic setup of the GC-NCI-qMS device, equipped with a gas chromatograph (GC), negative chemical ionization (NCI), and quadrupole mass spectrometry (qMS).

The working solutions were directly analyzed by GC-qMS (QP2010, Shimadzu, Tokyo, Japan) coupled with a negative chemical ionization (NCI) source and an AOC-20i autosampler (Figure 1). For each working solution, five replicated injections were consecutively carried out in a batch. An HP-5MS capillary column (30 m length, 0.25 mm diameter, and 0.25 *μ*m film thickness, Agilent Technologies) was used to separate the mixture. Helium was used as the carrier gas with a constant flow of 1.00 mL min^−1^. Splitless injection mode was selected with splitless time of 1 min, and the injection volume was 1 *μ*L with the injector temperature maintained at 270°C. The oven program was set as follows: initial temperature of 60°C, held for 2.0 min, then ramped to 300°C at 25°C min^−1^ and held for 2.0 min.

For the conditions of qMS, the ion source and interface temperatures were maintained at 240 and 260°C, respectively. The ionization energy (IE) and emission current (EC) were set at 90 eV and 90 *μ*A, respectively (see Section 3). Pure methane (>99.999%) was used as the reactant gas at a pressure of 0.2 MPa. Full scan mode (SCAN) was used to monitor all ionization fragments in the range of *m*/*z* 30-360 to determine the fragmentation pattern. Single ion monitoring (SIM) recorded only two mass peaks, at *m*/*z* 35 and 37 (corresponding to ^35^Cl^−^ and ^37^Cl^−^), to acquire the ^37^Cl/^35^Cl ratio. The recorded mass span was set to 0.1 mass units. To avoid interference, the quadrupole mass spectrometer was tuned with reference gas tris (perfluorobutyl) amine to optimize low and high mass resolution parameters and obtain optimal isotopic resolution. Data were recorded using GC/MS Solution Version 2.50SU1 software provided by Shimadzu (Japan).

### 2.4. Calculation of Chlorine Isotope Ratios

Peak identification was performed by analyzing the slopes of the mass traces following established procedures for critical peak detection and integration parameters. The peak start was defined as the retention time when the slope exceeded a threshold value, while the peak end was defined as the retention time when the slope passed the threshold value after reaching its minimum. In this study, the slope threshold value was set at 2% in the GC/MS solution software. More details about peak identification could be found in references [20, 25, 26] and supplemental materials within them. The peak area was calculated as a sum of all the intensities between the beginning and the end of a peak.

Chlorine isotope ratios (IR) were directly calculated from the peak areas at *m*/*z* 37 and *m/z* 35. Therefore, the relative isotope ratio variation (*δ*^37^Cl′) was calculated as follows:

(1) $$\begin{array}{c} \text{I}{\text{R}}_{\text{Cl}}=\frac{A\left({\;}^{37}\text{Cl}\right)}{A\left({\;}^{35}\text{Cl}\right)}, \end{array}$$

(2) $$\begin{array}{c} \;\delta^{37}\text{C}{\text{l}}^{\prime }=\left(\frac{\text{I}{\text{R}}_{\text{sam}}}{\text{I}{\text{R}}_{\text{std}}}-1\right)\times 1000‰, \end{array}$$

where A (^37^Cl) and A (^35^Cl) are the peak areas at *m/z* 37 and 35, and IR_sam_ and IR_std_ are the chlorine IRs (^37^Cl^−^/^35^Cl^−^) of the sample and standard, respectively.

As organochlorine isotope standards are unavailable until now, and AccuStandard was used as the external isotope standards for the calibration of *δ*^37^Cl′. Consequently, the *δ*^37^Cl′ reported in this study is relative to the ^37^Cl/^35^Cl value of AccuStandard standards, rather than standard mean ocean chlorine (SMOC) [27]. Actually, the standards purchased from different suppliers could be considered as mutual external isotope standards for each other.

## 3. Results and Discussion

### 3.1. Comparison of NCI and EI

According to the valence bond theory, the dissociation energy of C–Cl bonds is less than that of C–C bonds. Appropriate energy can dissociate C–Cl bonds rather than C–C bonds, separating Cl from the compound and producing pure Cl^−^ from some chlorinated organic compounds.

**Figure 2:**
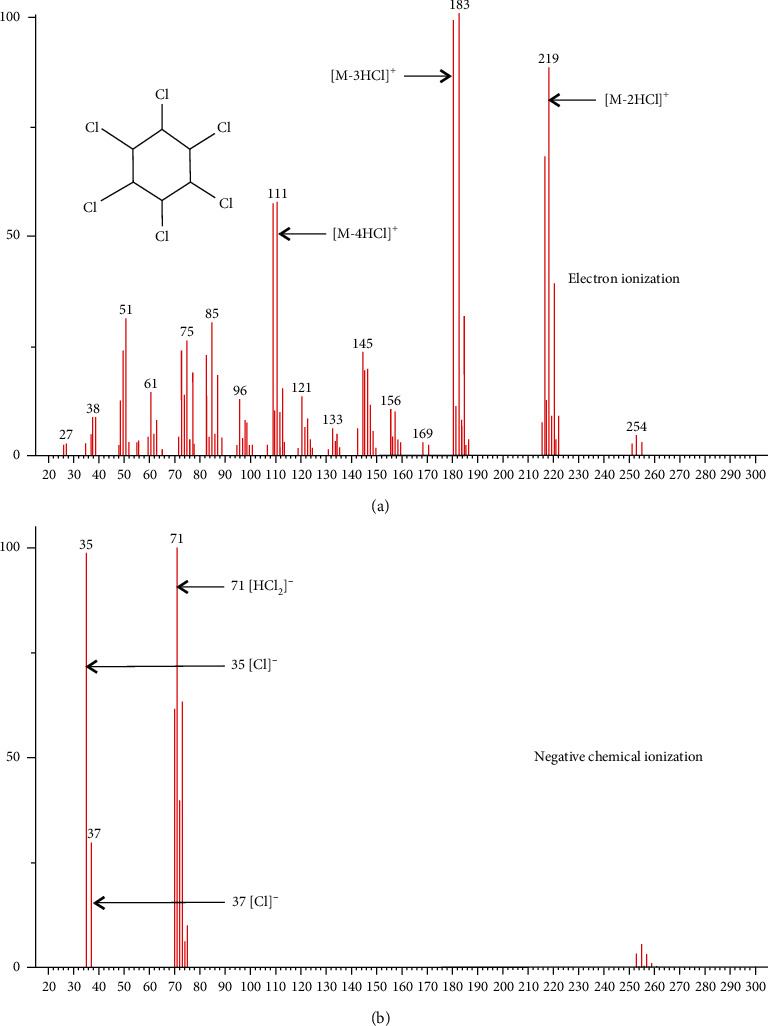
A comparison of mass spectrum from electron ionization (EI) and negative chemical ionization (NCI) of *α*-hexachlorocyclohexane (*α*-HCH). (a) EI mass spectrum extracted from NIST Database V 2.0; (b) NCI mass spectrum in SCAN mode obtained in this study.

Prior studies on CSIA-Cl methods have generally used GC-qMS [19–22], GC-QTOF-MS [28], or GC-DFS-HRMS [29]. However, these studies have all used electron ionization (EI), which produces many ion fragments. This resulted in weak peak signals for fragments containing chlorine and complex spectrum, which affected method precision and accuracy. For example, using EI, *α*-HCH produced weak molecular ion peaks and common fragments comprising a series of ions losing Cl and HCl (Figure 2(a)). Compared with EI, chemical ionization is a type of “soft” ionization that produces few compound fragments. In negative chemical ionization (NCI), the sample molecules do not directly interact with electrons emitted from the filament, but reaction gas molecules collide with the electrons to generate hot electrons, which are trapped by the sample molecules to generate negative ions, achieving ionization. NCI can detect compounds with strong electronegativity, such as organochlorines, that readily capture electrons, resulting in high sensitivity and selectivity [30]. The characteristic ions of *α*-HCH appeared at *m/z* 35 (Cl^−^) and 71 (HCl_2_^−^), while the relative abundance of ions in the high mass region was small (Figure 2(b)). The main ions and fragments of eight organochlorine pesticides from EI and NCI are listed in Table 1. Regarding the aforementioned merits of NCI, NCI was favored to carry out the experiments that follow.

### 3.2. Optimization of Ionization Energy (IE) and Emission Current (EC)

The abundance of ionized fragments depends on both IE and EC. Furthermore, IE also determines the species of the ionized fragments. The standard stock solution of eight organochlorine pesticides was measured in the IE range of 20–100 eV and EC range of 20–100 *μ*A under SCAN mode, respectively.

**Figure 3:**
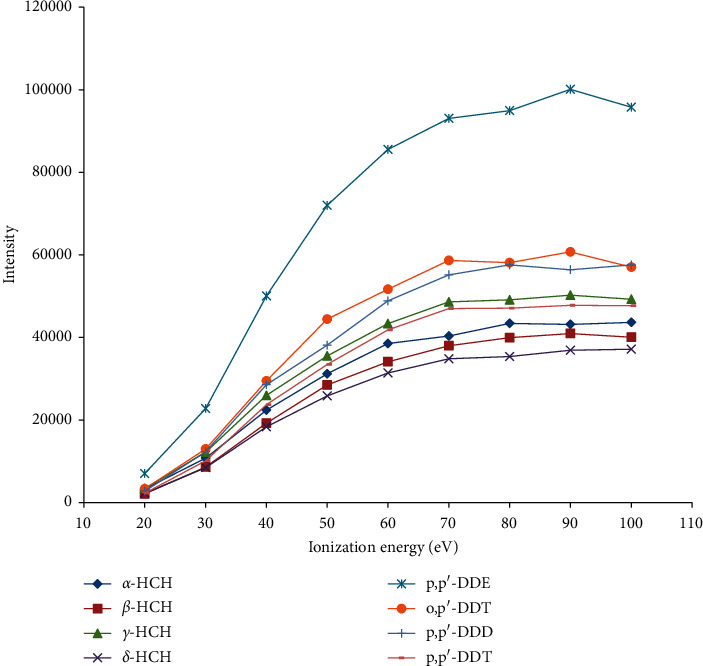
Absolute intensity of chloride ionized at different ionization energy values for eight organochlorine pesticides: *α*-hexachlorocyclohexane (*α*-HCH), *β*-hexachlorocyclohexane (*β*-HCH), *γ*-hexachlorocyclohexane (*γ*-HCH), *δ*-hexachlorocyclohexane (*δ*-HCH), 1-chloro-2-[2, 2, 2-trichloro-1-(4-chlorophenyl) ethyl] benzol (o, *p′*-DDT), 2, 2-bis (*p*-chlorophenyl)-1, 1, 1-trichloroethane (*p*, *p′*-DDT), 2, 2-dichloro-1, 1-bis (4-chlorophenyl) ethylene (*p*, *p′*-DDE), and 1, 1-dichloro-2, 2-bis (*p*-chlorophenyl) ethane (*p*, *p′*-DDD).

**Figure 4:**
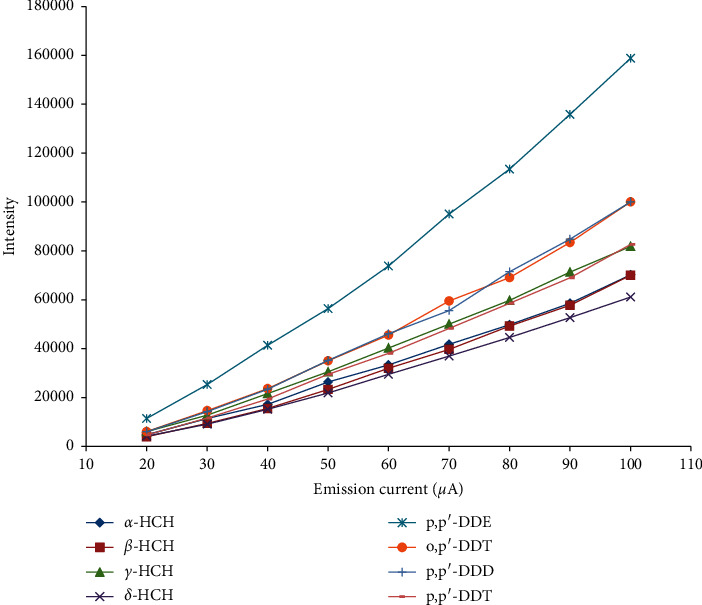
Absolute intensity of chloride ionized at different emission current values for eight organochlorine pesticides: *α*-hexachlorocyclohexane (*α*-HCH), *β*-hexachlorocyclohexane (*β*-HCH), *γ*-hexachlorocyclohexane (*γ*-HCH), *δ*-hexachlorocyclohexane (*δ*-HCH), 1-chloro-2-[2, 2, 2-trichloro-1-(4-chlorophenyl) ethyl] benzol (o, *p′*-DDT), 2, 2-bis (*p*-chlorophenyl)-1, 1, 1-trichloroethane (*p*, *p′*-DDT), 2, 2-dichloro-1, 1-bis (4-chlorophenyl) ethylene (*p*, *p′*-DDE), and 1, 1-dichloro-2, 2-bis (*p*-chlorophenyl) ethane (*p*, *p′*-DDD).

The intensity of the total ions as a function of IE variation is depicted in Figure 3. It illustrates that the intensity of the total ions gradually increases along with IE increasing until 70 eV and then reaches its plateau between 70 and 100 eV. For most of the organochlorine pesticides, the IE of maximum abundance is 90 eV. However, the intensity of the total ions continuously increases with EC increasing in the range of 20–100 *μ*A (Figure 4). The reason we have not tried the high EC (beyond 100 *μ*A) is that an excessive current will lead to high background and quickly deplete the filament within a very short timeframe. The intensity of the total ions obtained under 90 eV and 90 *μ*A is sufficient for us to determine the ^35^Cl/^37^Cl ratio. Therefore, with overall consideration, 90 eV and 90 *μ*A are regarded as the optimal experimental parameters. Notably, the isotope fractionation effect may exist in ionization processes of NCI, which could be corrected by the external isotope standards.

### 3.3. Evaluation of Different Analysis Modes

**Figure 5:**
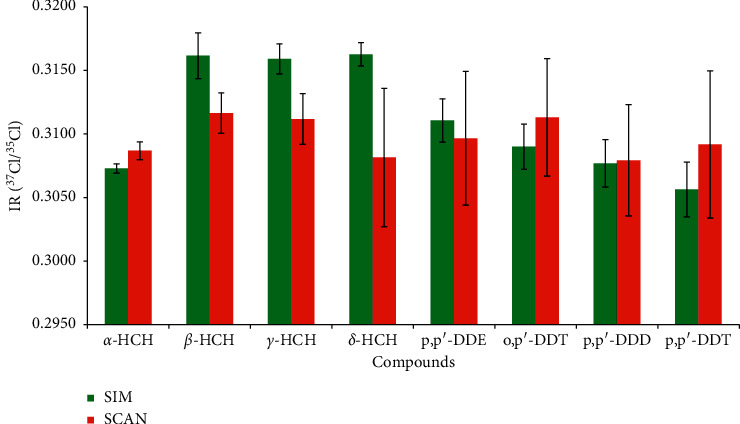
IR (^37^Cl/^35^Cl) values and precisions from single ion monitoring (SIM) and scan mode (SCAN). Error bars denote the standard deviations (1*σ*). Eight organochlorine pesticides: *α*-hexachlorocyclohexane (*α*-HCH), *β*-hexachlorocyclohexane (*β*-HCH), *γ*-hexachlorocyclohexane (*γ*-HCH), *δ*-hexachlorocyclohexane (*δ*-HCH), 1-chloro-2-[2, 2, 2-trichloro-1-(4-chlorophenyl) ethyl] benzol (o, *p′*-DDT), 2, 2-bis (*p*-chlorophenyl)-1, 1, 1-trichloroethane (*p*, *p′*-DDT), 2, 2-dichloro-1, 1-bis (4-chlorophenyl) ethylene (*p*, *p′*-DDE), and 1, 1-dichloro-2, 2-bis (*p*-chlorophenyl) ethane (*p*, *p′*-DDD).

GC/MS has two analysis modes, namely, SCAN and SIM. To assess the impact of other ions, the precision and accuracy of IRs were also evaluated using different analysis modes by extracting *m/z* 35 (Cl^−^) and 37 (Cl^−^) from SCAN mode. As shown in Figure 5, when five consecutive replicated injections were conducted, the resulting IRs were comparable in different modes and the standard deviation of *α*-HCH showed the highest precision of 0.37‰ under SIM mode. Compared with SCAN mode, most compounds achieved higher precision in the range of 0.37‰–2.15‰ under SIM mode. As an exception, SCAN mode was slightly better than SIM mode for *β*-HCH (SCAN, 1.59‰; SIM, 1.80‰). SIM mode was chosen for CSIA-Cl since the majority of ions gave much better response.

### 3.4. Effects of Injection Amounts

**Figure 6:**
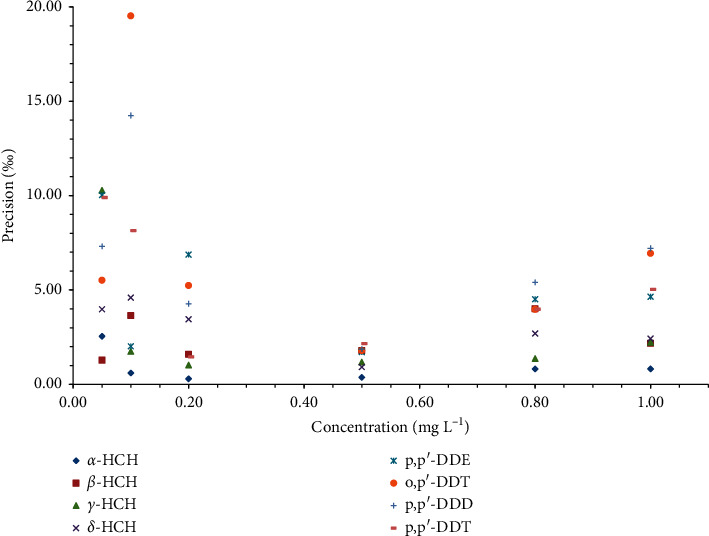
Precisions of IR (^37^Cl/^35^Cl) values at different concentrations for eight organochlorine pesticides: *α*-hexachlorocyclohexane (*α*-HCH), *β*-hexachlorocyclohexane (*β*-HCH), *γ*-hexachlorocyclohexane (*γ*-HCH), *δ*-hexachlorocyclohexane (*δ*-HCH), 1-chloro-2-[2, 2, 2-trichloro-1-(4-chlorophenyl) ethyl] benzol (o, *p′*-DDT), 2, 2-bis (*p*-chlorophenyl)-1, 1, 1-trichloroethane (*p*, *p′*-DDT), 2, 2-dichloro-1, 1-bis (4-chlorophenyl) ethylene (*p*, *p′*-DDE), and 1, 1-dichloro-2, 2-bis (*p*-chlorophenyl) ethane (*p*, *p′*-DDD).

**Figure 7:**
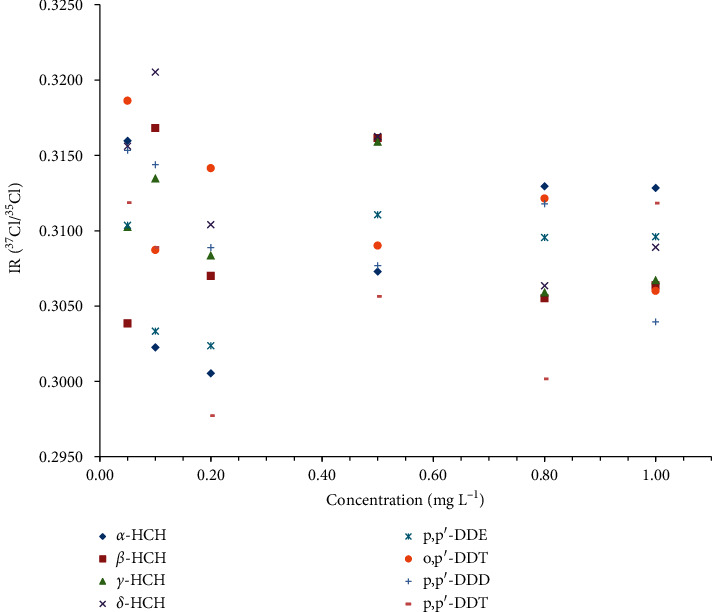
IR (^37^Cl/^35^Cl) values at different concentrations for eight organochlorine pesticides: *α*-hexachlorocyclohexane (*α*-HCH), *β*-hexachlorocyclohexane (*β*-HCH), *γ*-hexachlorocyclohexane (*γ*-HCH), *δ*-hexachlorocyclohexane (*δ*-HCH), 1-chloro-2-[2, 2, 2-trichloro-1-(4-chlorophenyl) ethyl] benzol (o, *p′*-DDT), 2, 2-bis (*p*-chlorophenyl)-1, 1, 1-trichloroethane (*p*, *p′*-DDT), 2, 2-dichloro-1, 1-bis (4-chlorophenyl) ethylene (*p*, *p′*-DDE), and 1, 1-dichloro-2, 2-bis (*p*-chlorophenyl) ethane (*p*, *p′*-DDD).

In previous studies, the amount of injected sample and isotope standard significantly affected IR precision and accuracy [20]. To evaluate the effects of injection amounts on the method accuracy and precision, different concentrations in the range of 0.05–1.00 mg L^−1^ were analyzed which adopted five replicated injections of the mixture. As shown in Figure 6, the precisions for the eight organochlorine pesticides were high, concomitantly with a narrow distribution in the range of 0.37‰–2.15‰ when the injection concentration was 0.50 mg L^−1^ (*viz*. 0.5 ng on column). However, high values with higher dispersion of precisions were obtained at other concentrations. For example, at the injection concentration of 0.10 mg L^−1^ (*viz*. 0.1 ng on column), the precision was 19.52‰ for *o*, *p′*-DDT and 14.24‰ for *p*, *p′*-DDD. The same tendency of concentration-dependent is obvious as indicated in Figure 7. Therefore, the ^37^Cl/^35^Cl ratio for the same analyte can be compared only for solutions of equal concentrations.

### 3.5. Application of the Novel Method

As mentioned above, the *δ*^37^Cl′ values of standards Supelco and O2si were calibrated to AccuStandard following Equation (2). The calculated *δ*^37^Cl′ values of Supelco and O2si are listed in Table 2. As shown in Table 2, the standard deviations of *δ*^37^Cl′ for *α*-HCH, *β*-HCH, *δ*-HCH, and *p*, *p′*-DDE (1.94‰–2.99‰) were lower than those of *γ*-HCH, *o*, *p′*-DDT, *p*, *p′*-DDD, and *p*, *p′*-DDT (3.44‰–6.76‰). Therefore, the *α*-HCH, *β*-HCH, *δ*-HCH, and *p*, *p′*-DDE in terms of precision are favorable for provenance identification by *δ*^37^Cl′.

**Figure 8:**
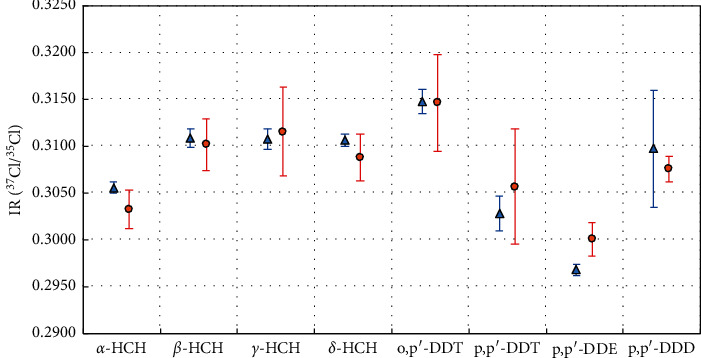
IR (^37^Cl/^35^Cl) values for standards from Supelco (blue) and O2si (red). For each compound, the bigger error bars denote three times maximum standard deviations and the smaller ones denote one time minimum standard deviations, respectively. Eight organochlorine pesticides: *α*-hexachlorocyclohexane (*α*-HCH), *β*-hexachlorocyclohexane (*β*-HCH), *γ*-hexachlorocyclohexane (*γ*-HCH), *δ*-hexachlorocyclohexane (*δ*-HCH), 1-chloro-2-[2, 2, 2-trichloro-1-(4-chlorophenyl) ethyl] benzol (o, *p*′-DDT), 2, 2-bis (*p*-chlorophenyl)-1, 1, 1-trichloroethane (*p*, *p′*-DDT), 2, 2-dichloro-1, 1-bis (4-chlorophenyl) ethylene (*p*, *p′*-DDE), and 1, 1-dichloro-2, 2-bis (*p*-chlorophenyl) ethane (*p*, *p′*-DDD).

To identify the provenance of samples, three times of the maximum standard deviations (3*δ*_max_) of IR values among candidates was regarded as the critical value [29]. According to statistical theory, if the discrepancy between two samples is larger than 3*δ*_max_, we could confidently distinguish them as from a different source. As shown in Figure 8 and Table 2, *α*-HCH and *p*, *p′*-DDE in samples (Supelco and O2si) are differentiated confidently, as IR of *α*-HCH in Supelco is completely larger than +3*δ*_max_ of that value in O2si, while IR of *p*, *p′*-DDE in Supelco is absolutely lower than -3*δ*_max_ of that value in O2si. Besides, two independent *T*-tests based on five *δ*^37^Cl*′* independent analyses (*viz.* five replications of injections) were performed to confirm the provenance identification by 3*δ*_max_. The probability of 0.003 within a 99.7% confidence interval was used as the critical value for the *T*-test. The probabilities of *T*-test are 0.000, 0.283, 0.386, 0.005, 0.000, 0.891, 0.088, and 0.045 for *α*-HCH, *β*-HCH, *γ*-HCH, *δ*-HCH, *p*, *p′*-DDE, *o*, *p′*-DDT, *p*, *p′*-DDD, and *p*, *p′*-DDT, respectively. Only the probabilities for *α*-HCH and *p*, *p′*-DDE are lower than the critical value of 0.003, which are identical to the judgment by the simple 3*δ*_max_ method. These two evaluations demonstrated that chlorine in *α*-HCH and *p*, *p′*-DDE from different companies was not homologous, while that in other compounds may have the same provenance. The method of provenance identification by *δ*^37^Cl′ value was well established with standards Supelco and O2si.

To validate the method in real samples, three organochlorine contaminated groundwater samples were analyzed. Sample 1 and sample 2 were collected from two adjacent polluted sites in the parts of the North China Plain in Shandong Province, while sample 3 was collected from another polluted site 50 km away. To minimize the effect of injection amounts, the sample concentration before injection should be adjusted to 0.50 mg L^−1^ (*viz*.0.5 ng on column) by dilution or enrichment during the sample pretreatment according to the measured concentration of organochlorine species. The *α*-HCH and *p*, *p′*-DDE in sample 1, *α*-HCH in sample 2, and *p*, *p′*-DDE in sample 3 were perceptible, whereas the concentrations of other organochlorine species were lower than limits of detection. As can be seen in Table 3, the negligible difference (probability of 0.917 > critical value of 0.003) in *δ*^37^Cl′ of *α*-HCH between sample 1 and sample 2 suggests that they have the same provenance. This result is in accordance with the fact that the two samples are collected from two adjacent polluted sites. The *δ*^37^Cl′ value for *p*, *p′*-DDE in sample 1 and sample 3 clearly indicates that significant difference (probability of 0.000 < critical value of 0.003) is present between sample 1 and sample 3, implying two independent sources for them.

## 4. Conclusions

In this study, the CSIA-Cl method for organochlorine pesticide analysis by GC-NCI-qMS was established. The simple mass spectrum generated by NCI was compared with EI. The optimal operational parameter was 90 eV for IE and 90 *μ*A for EC under SIM analysis mode with 0.50 mg L^−1^ (*viz*. 0.5 ng on column) injected concentration which adjusted well in terms of total ions intensity and precision of IRs (^35^Cl/^37^Cl). Under the optimized conditions, the precisions for eight organochlorine pesticides ranged from 0.37 ‰ to 2.15 ‰. It is worth noting that the highest precision for the ^37^Cl/^35^Cl ratio could be achieved only for solutions of equal concentrations due to its concentration-dependent effect. Furthermore, this novel CSIA-Cl method was validated using mutual external isotope standards and applied to the groundwater in the environment for provenance identification by *δ*^37^Cl′. Although the established method needs double determinations for samples of unknown concentration, it completely overcomes the shortcomings of isobaric interference and complicated correction formulas exhibited by other methods.

This method could, in principle, be applied to other organochlorines with strong electronegativity to obtain high sensitivity and selectivity. However, factors that may impact isotope fractionation and its degree, and the detailed cracking reaction mechanisms, need further investigation. The studies to develop methods to calibrate AccuStandard standard by SMOC are now underway.

## Figures and Tables

**Table 1:** Main ions and fragments of eight organochlorine pesticides obtained from electron ionization (EI) and negative chemical ionization (NCI).

Compounds	EI	NCI
HCH (*α, β, γ, δ*)	[M-nHCl]^+^, [M-nCl]^+^	35 [Cl]^−^, 71 [HCl_2_]^−^, 37 [Cl]^−^
DDT (*o*, *p′*-, *p*, *p′*-)	235 [(C_6_H_4_Cl)_2_CH]^+^	35 [Cl]^−^, 71 [HCl_2_]^−^, 37 [Cl]^−^
*p*, *p′*-DDE	318 [M]^+^, 246[M-2HCl]^+^	35 [Cl]^−^, 37 [Cl]^−^
*p*, *p′*-DDD	235 [(C_6_H_4_Cl)_2_CH]^+^	35 [Cl]^−^, 71 [HCl_2_]^−^, 37 [Cl]^−^

Note: M represents molecular ion; eight organochlorine pesticides: *α*-hexachlorocyclohexane (*α*-HCH), *β*-hexachlorocyclohexane (*β*-HCH), *γ*-hexachlorocyclohexane (*γ*-HCH), *δ*-hexachlorocyclohexane (*δ*-HCH), 1-chloro-2-[2, 2, 2-trichloro-1-(4-chlorophenyl) ethyl] benzol (*o*, *p′*-DDT), 2, 2-bis (*p*-chlorophenyl)-1, 1, 1-trichloroethane (*p*, *p′*-DDT), 2, 2-dichloro-1, 1-bis (4-chlorophenyl) ethylene (*p*, *p′*-DDE), and 1, 1-dichloro-2, 2-bis (*p*-chlorophenyl) ethane (*p*, *p′*-DDD).

**Table 2:** Isotope ratios (IR (^37^Cl/^35^Cl)), standard deviations (1*σ*), and *δ*^37^Cl′ for Supelco standards and O2si standards calculated in relation to external isotope standards from AccuStandard.

Compounds	AccuStandard (IR (^37^Cl/^35^Cl), 1*σ*)	Supelco (IR (^37^Cl/^35^Cl), 1*σ*)	O2si (IR (^37^Cl/^35^Cl), 1*σ*)	Supelco *δ*^37^Cl′ (1*σ*, %)	O2si *δ*^37^Cl′ (1*σ*, %)
*α*-HCH	0.30464 ± 0.00032	0.30551 ± 0.00059	0.30314 ± 0.00069	2.86 ± 1.94	−4.91 ± 2.26
*β*-HCH	0.31069 ± 0.00041	0.31081 ± 0.00093	0.31014 ± 0.00093	0.39 ± 2.99	−1.78 ± 2.98
*γ*-HCH	0.31014 ± 0.00075	0.31072 ± 0.00107	0.31150 ± 0.00158	1.89 ± 3.44	4.41 ± 5.08
*δ*-HCH	0.31050 ± 0.00073	0.31062 ± 0.00065	0.30876 ± 0.00084	0.40 ± 2.09	−5.59 ± 2.72
*o*, *p′*-DDT	0.31560 ± 0.00113	0.31473 ± 0.00129	0.31459 ± 0.00173	−2.78 ± 4.10	−3.22 ± 5.48
*p*, *p′*-DDT	0.30595 ± 0.00146	0.30273 ± 0.00182	0.30562 ± 0.00204	−10.55 ± 5.96	−1.09 ± 6.66
*p*, *p′*-DDE	0.29923 ± 0.00105	0.29672 ± 0.00060	0.30002 ± 0.00060	−8.37 ± 2.02	2.63 ± 2.00
*p*, *p′*-DDD	0.30860 ± 0.00106	0.30967 ± 0.00209	0.30749 ± 0.00140	3.49 ± 6.76	−3.60 ± 4.54

Note: results were acquired from five replicated injections. Eight organochlorine pesticides: *α*-hexachlorocyclohexane (*α*-HCH), *β*-hexachlorocyclohexane (*β*-HCH), *γ*-hexachlorocyclohexane (*γ*-HCH), *δ*-hexachlorocyclohexane (*δ*-HCH), 1-chloro-2-[2, 2, 2-trichloro-1-(4-chlorophenyl) ethyl] benzol (o, *p*′-DDT), 2, 2-bis (*p*-chlorophenyl)-1, 1, 1-trichloroethane (*p, p*′-DDT), 2, 2-dichloro-1, 1-bis (4-chlorophenyl) ethylene (*p, p*′-DDE), and 1, 1-dichloro-2, 2-bis (*p*-chlorophenyl) ethane (*p, p′*-DDD).

**Table 3:** Results of groundwater samples.

Compounds	Sample-1 *δ*^37^Cl′ (1*σ*, ‰)	Sample-2 *δ*^37^Cl′ (1*σ*, ‰)	Sample-3 *δ*^37^Cl′ (1*σ*, ‰)
*α*-HCH	4.45 ± 1.22	4.54 ± 1.32	—
*p*, *p′*-DDE	−6.77 ± 2.09	—	2.95 ± 1.70

Note: the analytical concentration of samples was 0.50 mg L^−1^ (diluted or concentrated). Results were acquired from five replicated injections. Compounds: *α*-hexachlorocyclohexane (*α*-HCH) and 2, 2-dichloro-1, 1-bis (4-chlorophenyl) ethylene (*p*, *p*′-DDE).

## Data Availability

The data used to support the findings of this study are available from the corresponding author upon request.
